# Morphology, Structure and Function Characterization of PEI Modified Magnetic Nanoparticles Gene Delivery System

**DOI:** 10.1371/journal.pone.0098919

**Published:** 2014-06-09

**Authors:** Xiang Zhao, Haixin Cui, Wenjie Chen, Yan Wang, Bo Cui, Changjiao Sun, Zhigang Meng, Guoqiang Liu

**Affiliations:** 1 Institute of Environment and Sustainable Development in Agriculture, Chinese Academy of Agricultural Sciences, Beijing, China; 2 Nano biological Research Center, Chinese Academy of Agricultural Sciences, Beijing, China; 3 Biotechnology Research Institute, Chinese Academy of Agricultural Sciences, Beijing, China; University of Helsinki, Finland

## Abstract

Modified magnetic nanoparticles are used as non-viral gene carriers in biological applications. To achieve successful gene delivery, it is critical that nanoparticles effectually assemble with nucleic acids. However, relatively little work has been conducted on the assemble mechanisms between nanoparticles and DNA, and its effects on transfection efficiency. Using biophysical and biochemical characterization, along with Atomic force microscopy (AFM) and Transmission electron microscopy (TEM), we investigate the morphologies, assembling structures and gene delivering abilities of the PEI modified magnetic nanoparticles (MNPs) gene delivery system. In this gene delivery system, MNP/DNA complexes are formed via binding of DNA onto the surface of MNPs. MNPs are favorable to not only increase DNA concentration but also prevent DNA degradation. Magnetofection experiments showed that MNPs has low cytotoxicity and introduces highly stable transfection in mammalian somatic cells. In addition, different binding ratios between MNPs and DNA result in various morphologies of MNP/DNA complexes and have an influence on transfection efficiency. Dose–response profile indicated that transfection efficiency positively correlate with MNP/DNA ratio. Furthermore, intracellular tracking demonstrate that MNPs move though the cell membranes, deliver and release exogenous DNA into the nucleus.

## Introduction

Nanoparticles are widely used in gene therapy, drug delivery and diagnosis detection [1]–[5] due to its small size, surface effect and penetration performance. In recent years, magnetic nanoparticles have become one of the most popular nano-carriers, which have many benefits. Such benefits include high carrying efficiency [6] without inducing immunogenicity [7], it is biocompatible in living cells [8], and it is easy to design, modify and operate [9], [10]. Furthermore, magnetic nanoparticles exhibit paramagnetism, enabling high targeting in a magnetic field, thus increasing the intake of nucleic acids, transfection efficiency, and improving localization of a nucleic acid delivered to a specific area which is under the influence of a magnetic field [11]–[13].

The size, shape, surface charge and the presence of different modified functional groups of nanoparticles can affect cell-specific internalization, excretion, toxicity and efficacy [14]–[19]. It has been suggested that the optimal particle size for a targeting nano-carrier is 20–200 nm, which is large enough to avoid filtration but small enough to penetrate through the cell membrane [20]. The positively charged nanoparticles allow them to assemble with negatively charged phosphate backbone of nucleic acids due to electrostatically induced aggregation [13], [21]. The exposed charge of nanoparticles significantly affects their ability to internalize- and positively charged nanoparticles internalize rapidly via the clathrin-mediated pathway [22], [23]. However, after cell internalization, the intracellular trail and fate of nanoparticles has not yet been elucidated [24].

In order to achieve gene delivery, magnetic nanoparticles need to assemble with nucleic acids to form a nanoparticle/nucleic acid complex [13]. Effective complex formation is essential for exogenous gene delivery, release and expression, which are dependent on vectors or nucleic acids concentration, assembling ratio and assembling mechanism. There are two main assembly mechanisms, 1) adsorption [25] and 2) encapsulation [26], depending on vector size or modified functional groups.

In this study, we investigate the performance of PEI modified magnetic nanoparticles (MNPs) as gene vector, which are composed of Fe_3_O_4_ nanoparticles modified with branched polyethylenimine (PEI) [27], [28]. We characterize the biophysical properties and assembling morphologies of MNP/DNA complexes as well as evaluate the protection on exogenous DNA and cytotoxicity of MNP/DNA complexes. We successfully deliver enhanced green fluorescent protein (EGFP) plasmid pEGFP-N1 to porcine kidney 15 (PK 15) cells. The intracellular traces of MNP/DNA complexes are shown.

## Methods

### 2.1 Materials

The pEGFP-N1 plasmid expressed enhanced green fluorescent protein, was purchased from BD Biosciences Clontech (Palo Alto, CA, USA). PEI modified magnetic nanoparticles (MNPs) and red fluorescent MNPs were purchased from Chemicell (Berlin, Germany). Porcine kidney 15 (PK15) cell line was purchased from National platform of experimental cell resources for sci-tech (Beijing, China). Agarose gel was purchased from Biowest (Spain). *Hind* III, *DNase*|and MTT (3-(4,5-dimethylthiazolyl-2)-2, 5-diphenyltetrazolium bromide) were purchased from Sigma-Aldrich (Shanghai, China). 24 well plate, 96 well plate, DMEM and fetal bovine serum (FBS) were purchased from Gibco (Germany). Lipofectamine 2000 was purchased from Invitrogen (Germany). Gene Finder was purchased from BioV. Co. Ltd. (Xiamen, China). DAPI staining solution was purchased from Beyotime (Shanghai, China)

### 2.2 MNP/DNA complexes

MNPs and pEGFP-N1 plasmids were diluted with distilled water, mixed and incubated for 30 min at room temperature to form MNP/DNA complexes.

### 2.3 Transmission electron microscopy image

The MNPs or MNP/DNA solution prepared as described above were transferred onto hydrophilic-treated grids, then rinsed with ethanol and distilled water. Excess water was wicked off with filter paper. The grids were stained with 2% buffered phosphotunstic acid solution, and left overnight for complete dryness before the samples were imaged by transmission electron microscope [29] (TEM) (Philips CM120, Netherlands).

### 2.4 Atomic force microscopy image

Prior to atomic force microscopy (AFM) measurement (Mutimode 8, Bruker, USA), the suspensions were diluted with distilled water. 10 µl diluted solution was deposited onto a freshly cleaved mica substrate. Operating was performed in ScanAsyst-Air mode, using an Al reflective coating silicon nitride tip (Bruker, USA). All images were recorded in air at room temperature, at a scan speed of 0.99 Hz. The background slope was resolved using first or second order polynomial functions [30].

### 2.5 Size and Zeta potential measurements

Materials were dispersed in distilled water at the concentration of 20 µg/ml. Measurements were performed at 25°C, using a laser particle size analyzer (Zetasizer Nano ZS90, Malvern, England).

### 2.6 Combination and protection analysis of MNP/DNA complexes

Pure plasmid DNA and MNP/DNA complexes with different mass ratios were run on a 0.6% (w/v) agarose gel for 40 min at 100V. For protection analysis, pure plasmid DNA and MNP/DNA complexes were incubated in the presence of 1 U *Hind* III and *DNase*|respectively at 37°C, digested for 16 h. The final DNA weight of pure plasmid and all MNP/DNA complexes load on the gel were adjusted to 1 µg. Fluorescent MNPs labeled with Lumogen F Red 305 were used. DNA was stained with Gene Finder. Gel was scanned on the Typhoon 9400 scanner (Amersham Biosciences, USA).

### 2.7 Magnetofection in PK15 cells

PK 15 cells were seeded in a 24 well plate with a density of 10^6^ cells/well, and grown for 24 h until they reached 70%–80% confluence. After rinsed twice with PBS and DMEM without serum or other supplement, cells were incubated with MNP/DNA complexes on a 0.3 T permanent magnet for 4 h. In details, Group a: 10^6^ cells treated with 0.25 µg MNPs in MNP/DNA mass ratio of 1∶1, 1∶2, and 1∶4 respectively. Group b: 10^6^ cells treated with 0.5 µg MNPs with MNP/DNA mass ratio of 1∶1, 1∶2, and 1∶4 respectively. Group c: 10^6^ cells treated with 1 µg MNPs with MNP/DNA mass ratio of 1∶1, 1∶2, and 1∶4 respectively. Group d: cells treated with lipofectamine. The control group was treated with plasmid DNA. Then medium was replaced with DMEM supplemented with 10% FBS and cultured at 37°C, 5% CO_2_.

To track the intracellular traces, red fluorescent MNPs were used. Cells were treated following the method described above. After magnetofection, cell nucleuses were labeled with DAPI staining solution. The expression of EGFP gene and intracellular traces of MNPs were observed by inverted fluorescence microscope (Olympus IX71, Japan) with time.

### 2.8 Cytotoxicity test

PK15 cells were seeded in a 96 well plate with a density of 10^4^ cells/well and cultured at 37°C, 5% CO_2_ for 24 h. Then PK15 cells were treated by MNP/DNA complexes with different concentrations and lipofectamine respectively. PK15 cells untreated were used as control. After incubation for 6 h, the medium was removed. PK15 cells were cultured at 37°C, 5% CO_2_ for 24 h, and were cultured for another 4 h in MTT (0.5%) containing DMEM, then the medium was carefully removed. 150 µl/well dimethyl sulfoxide was added and oscillated gently to make crystal dissolved. The absorbance at 490 nm was measured using a microplate reader. The cell viability was expressed as a percentage of OD490 (sample)/OD490 (control).

## Results and Discussion

### 3.1 Characterization of PEI modified magnetic nanoparticles

**Figure 1:**
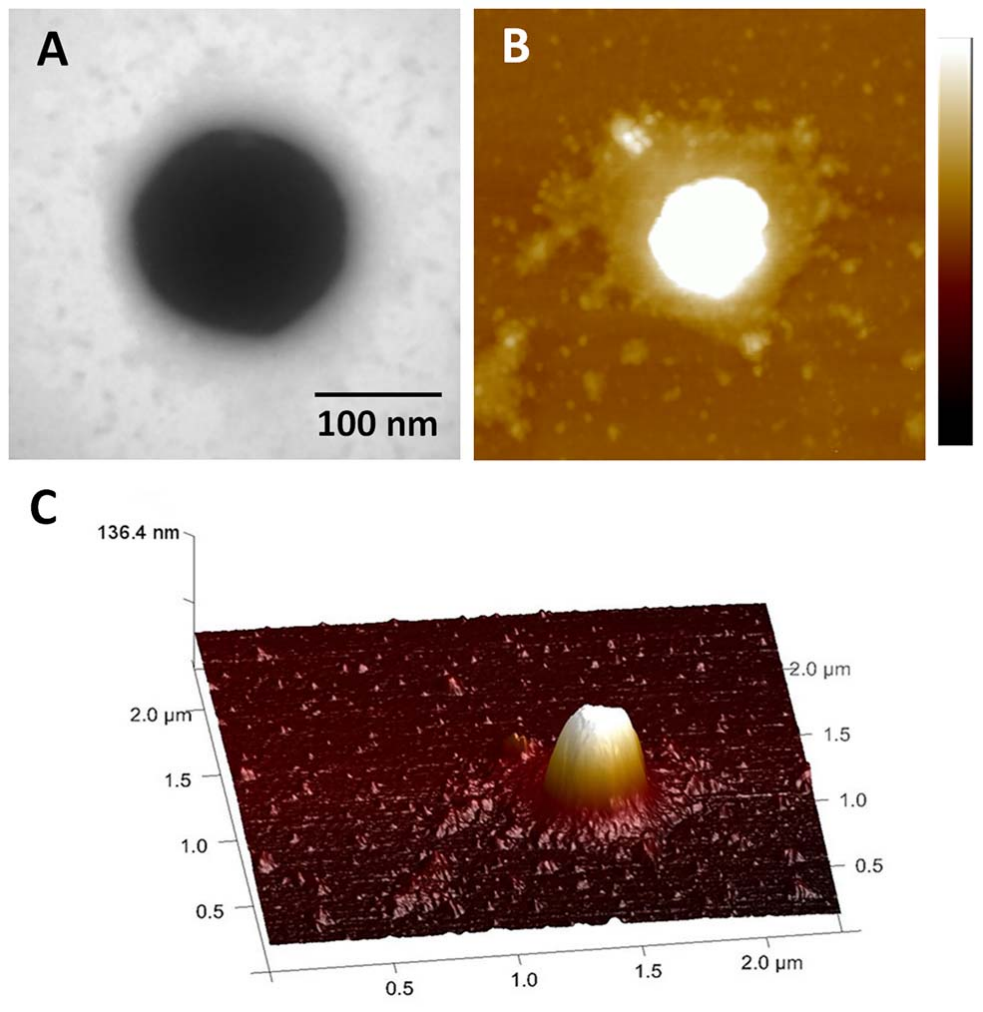
TEM image and AFM images of MNPs. **A**) TEM image. **B**) AFM height image (scan size  = 2 µm scale bar  = 100 nm). **C**) 3D rending AFM image.

The morphologies of MNPs were characterized by TEM and AFM. Core-shell structure of MNP was clearly identified in the magnified image of a single MNP (Fig. 1A). Core phase consisted of Fe_3_O_4_ nanoparticle showed dark central image comparing with the shells of PEI polymer. The PEI shell could effectively absorb DNA via electrostatic attraction. Same structure was also observed by AFM (Fig. 1B). Fig. 1C showed a 3D-AFM-image of a single MNP on the mica substrate with 136.4 nm height, which was the optimal particle size for targeting nano-carriers that illustrated in previous studies [14].

### 3.2 Size and zeta potential measurements

**Figure 2:**
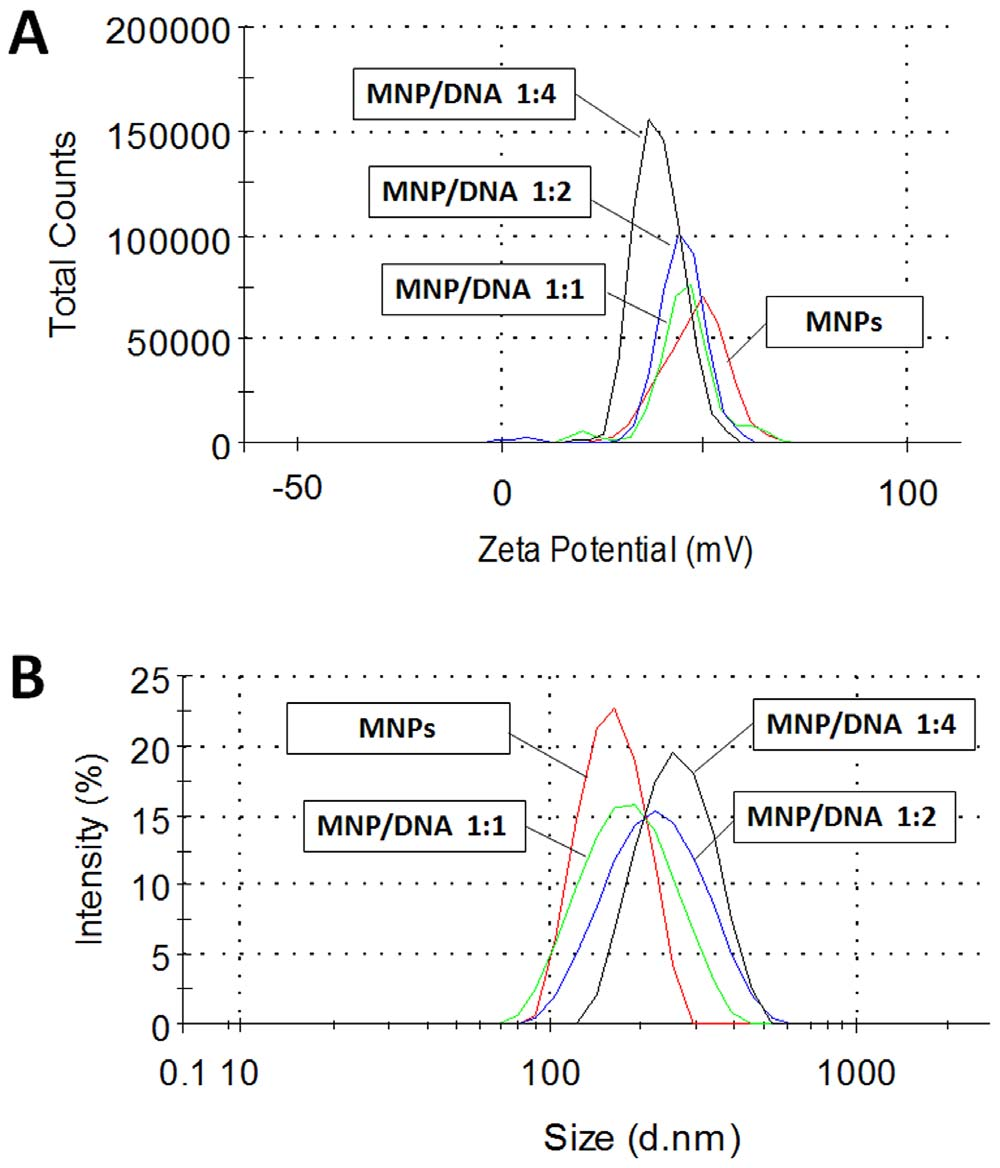
Size and zeta potential assay of MNPs and MNP/DNA complexes. The average diameter gradually increased (A) and the zeta potential gradually decreased (B) as the ratio of conjugated DNA increased, the values are shown in Table 1.

In order to evaluate the DNA-binding ability of MNPs as gene vector, the hydrodynamic diameter and surface charge measurements of MNPs and MNP/DNA complexes were conducted. MNPs were 168±3.2 nm in diameter with positive charge of +48.2±0.6 mV. The MNP/DNA complexes showed the increased average diameter and the decreased zeta potential due to due to the combination with electric negative DNA (Fig. 2 and Table 1). These results suggested that MNPs had effectively been loaded with DNA. The excess positive charge of MNP/DNA complexes was advantageous to the accumulation on the cell membrane and facilitating intracellular uptake [22].

**Table 1:** Size and Zeta potential assay of MNPs and MNP/DNA complexes.

	Average diameter (nm)	Zeta potential (mV)
**MNPs**	168±3.2	+48.2±0.6
**MNP/DNA complexes (mass ratio 1∶1)**	182±2.6	+45.6±2.3
**MNP/DNA complexes (mass ratio 1∶2)**	212±1.4	+42.2±1.2
**MNP/DNA complexes (mass ratio 1∶4)**	326±2.5	+38.8±1.4

### 3.3 Assembling mechanism of MNP/DNA complexes

**Figure 3:**
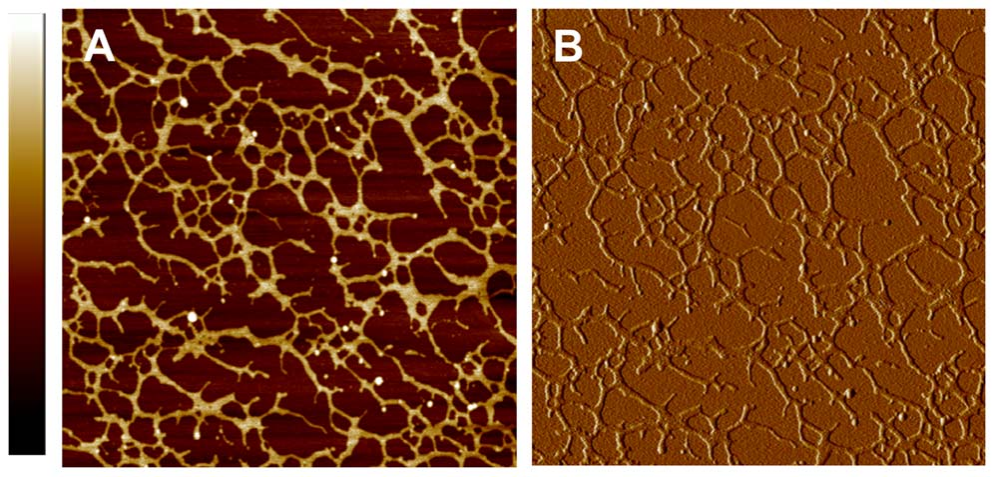
AFM images of plasmid DNA. **A**) Height image (scan size  = 5 µm, scale bar  = 4 nm). **B**) Corresponding peak force error image.

To investigated the mechanism of MNPs assembling with DNA, the morphologies of plasmid DNA and MNP/DNA complexes were characterized by AFM. DNA molecules distributed on the substrate with displaying a closed geometry like a network (Fig. 3). This structure has been reported in uncondensed DNA morphology [31].

**Figure 4:**
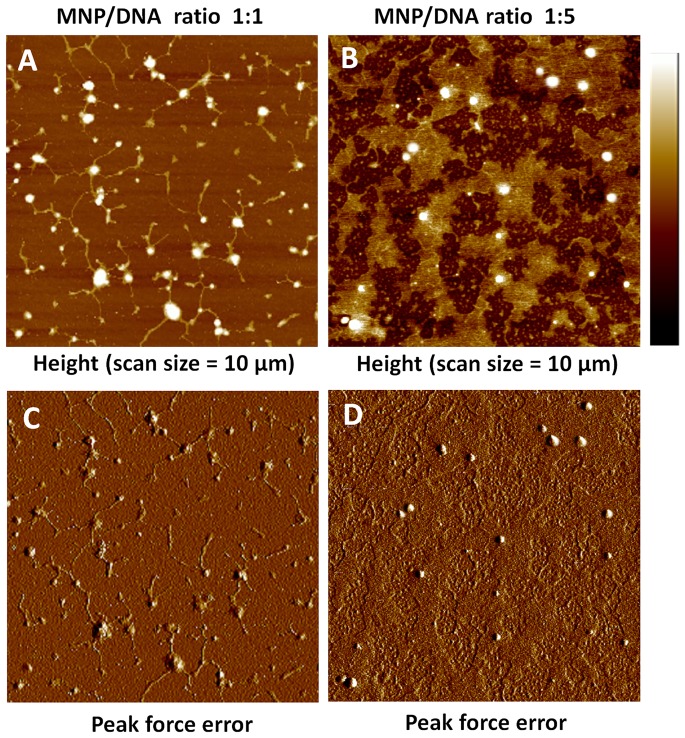
AFM images of MNP/DNA complexes. **A**) and **B**) Height images of MNP/DNA complexes with mass ratio 1∶1 and 1∶5 (scan size  = 10 µm, scale bar  = 100 nm). **C**) and **D**) Corresponding peak force error images.

**Figure 5:**
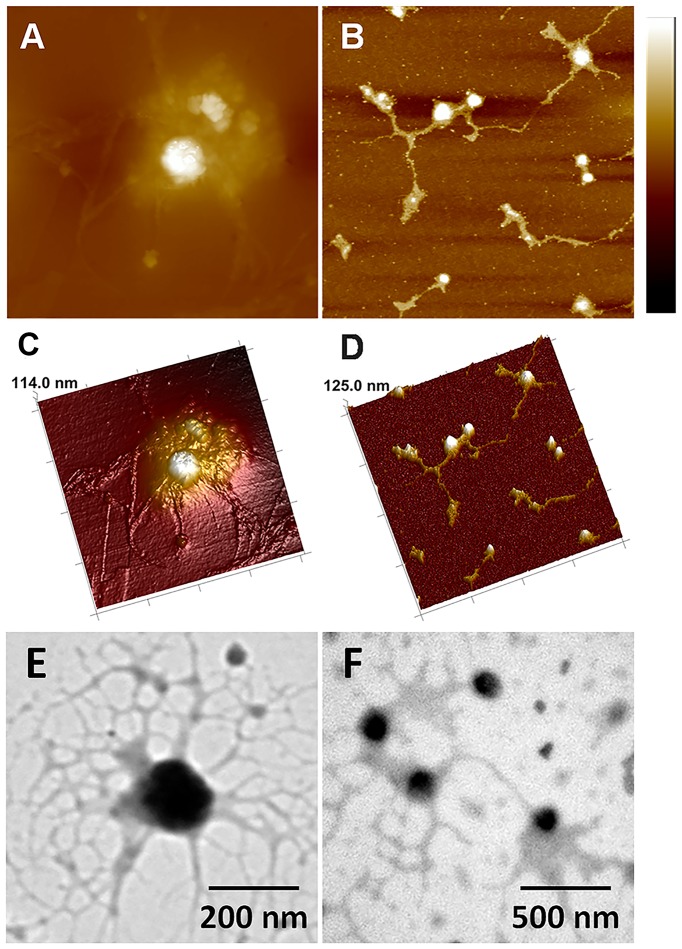
AFM and TEM images of MNP/DNA complexes. **A**) and **B**) AFM height images (scan size  = 1.5 µm and 2 µm, scale bar  = 100 nm). **C**) and **D**) Corresponding 3D images. **E**) and **F**) TEM images of MNP/DNA complexes.

The morphologies of MNP/DNA complexes with different mass ratios were shown in Fig. 4. The well-condensed structures confirmed the adsorption of DNA molecules on the surface of the nanoparticles [32]. When the complexes were formed with the MNP/DNA ratio of 1∶1, the plasmid DNA molecules were adsorbed and aggregated on the surface of the nanoparticles tightly, resulting in rod-shaped structures of DNA molecules [33] (Fig. 4A, C). As the ratio of DNA increased (MNP/DNA 1∶5, Fig. 4B, D), the adsorbed DNA molecules increased and arranged radially centering on the nanoparticles, which were distributed like “islands”. These results were consistent with the morphology analysis of TEM image (Fig. 5).

The AFM and TEM results visually demonstrated that MNPs acted as linkers for DNA molecules and effectively adsorbed DNA molecules on their surface, resulting in the formation of MNP/DNA complexes, which is favorable to delivery gene into cells by cell endocytosis.

### 3.4 Protection analysis of magnetic nanoparticles for plasmid DNA

**Figure 6:**
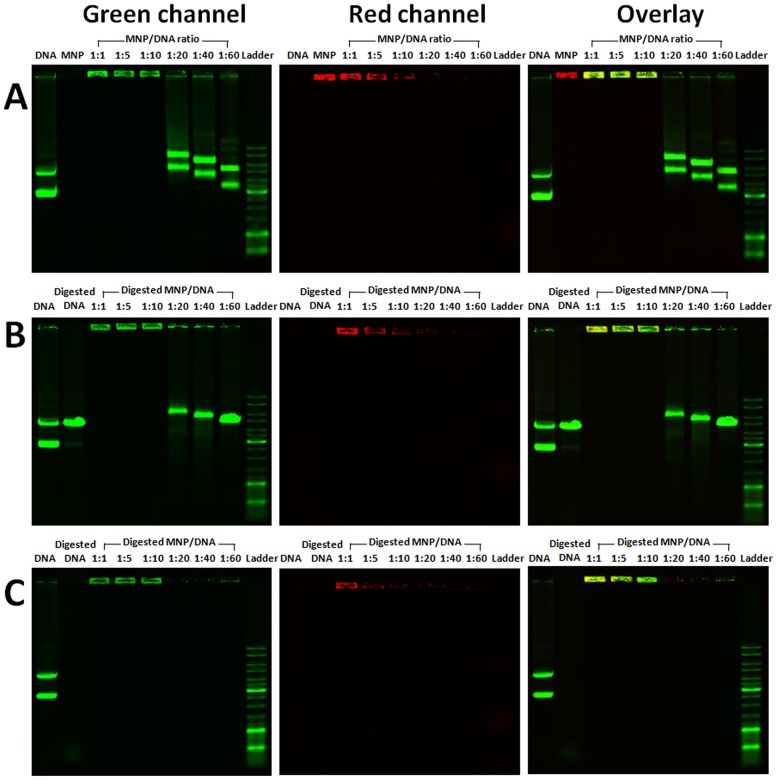
Agarose gel electrophoresis of plasmid DNA and MNP/DNA complexes. Co-migration of MNP (red) and DNA (green) on gel (A). Plasmid DNA and MNP/DNA complexes were digested with DNase I (B), and Hind III(C).

Complexation between the MNP and DNA was further confirmed by agarose gel electrophoresis. Co-migration of MNP (red) and DNA (green) revealed that MNP effectively complexed DNA (Fig. 6A). MNP/DNA complexes (yellow) retained at wells due to the increased size of the complexes. A distinct band shift of exceed DNA was also observed at the mass ratio of 1∶20, 1∶40 and 1∶60. In addition, MNP/DNA complexes were digested with endonuclease *DNase* I (Fig. 6B) and restriction enzymes *Hind* III (Fig. 6C). Lack of smear indicated that MNP/DNA complexes can protect DNA against degradation and retain in wells. As the concentration of MNPs decreases, exceed DNA are digested. Digestion results verified the protecting function of MNPs against enzymatic degradation, which was vital to accomplish higher gene transfection efficiency.

### 3.5 Expression efficiency of magnetofection

**Figure 7:**
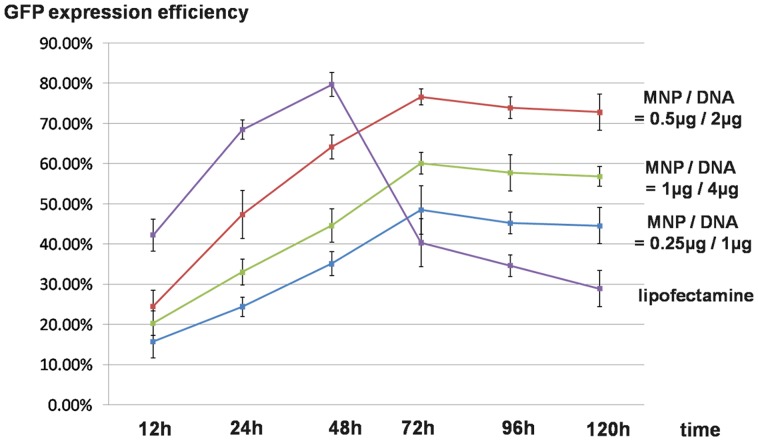
GFP expression efficiency of magnetofection and liposome transfection in PK15 cells with time.

EGFP expression plasmid (pEGFP-N1) was successfully delivered to PK15 cells by MNPs under the optimized experimental conditions. Compared with traditional lipofectamine transfection reagent, cells treated with MNPs achieved stable transfection and long-term effects of exogenous DNA expression (Fig. 7). The GFP expression efficiency of lipofectamine decreased sharply (40.32%) after 48 h. Cells treated with MNP/DNA complexes kept a sustained growth in GFP expression and reached the peak at 72 h (76.60%, MNP/DNA 0.5 µg/2 µg). The expression efficiency still kept a high level of 72.3% (MNP/DNA 0.5 µg/2 µg) after 120 h, which indicated stable expressions of MNPs as gene vector.

### 3.6 Dose–response profile

**Figure 8:**
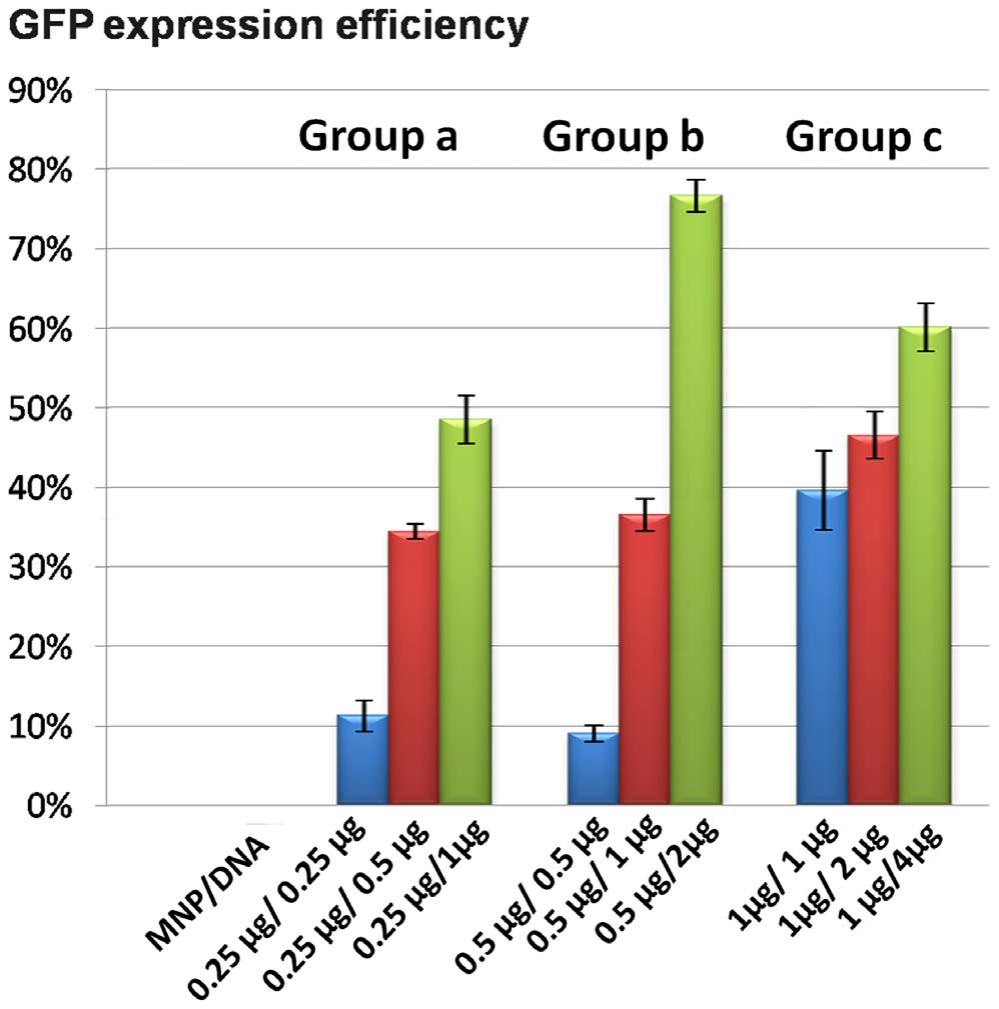
Dose–response relationships of GFP expression in PK15 cells with time. Group a: 106 cells treated with 0.25 µg MNPs with MNP/DNA mass ratio of 1∶1, 1∶2, 1∶4 respectively. Group b: 106 cells cells treated with 0.5 µg MNPs with MNP/DNA mass ratio of 1∶1, 1∶2, and 1∶4 respectively. Group c: 106 cells treated with 1 µg MNPs with MNP/DNA mass ratio of 1∶1, 1∶2, and 1∶4 respectively.

The efficiency of exogenous gene expression was influenced not only by the dose of gene vectors or exogenous DNA but also by the assemble ratio (Fig. 8). Exogenous gene expression efficiency increased as the MNP/DNA ratio decreased. When the MNP/DNA ratio decreased to 1∶4, it resulted in a synergistic improvement of dose–response relationships. The expressing efficiency was decreased with the lower MNP/DNA ratio at 1∶1. The reason is that the DNA is difficult to release from MNP/DNA complex, resulting in the expressing efficiency and low dose–response profile. As shown in AFM characterization (Fig. 4A), DNA were absorbed tightly by MNPs. In addition, the exceeded MNPs without bounding to DNA may induce cytotoxicity to the cells.

### 3. 7 Cytotoxicity test

**Figure 9:**
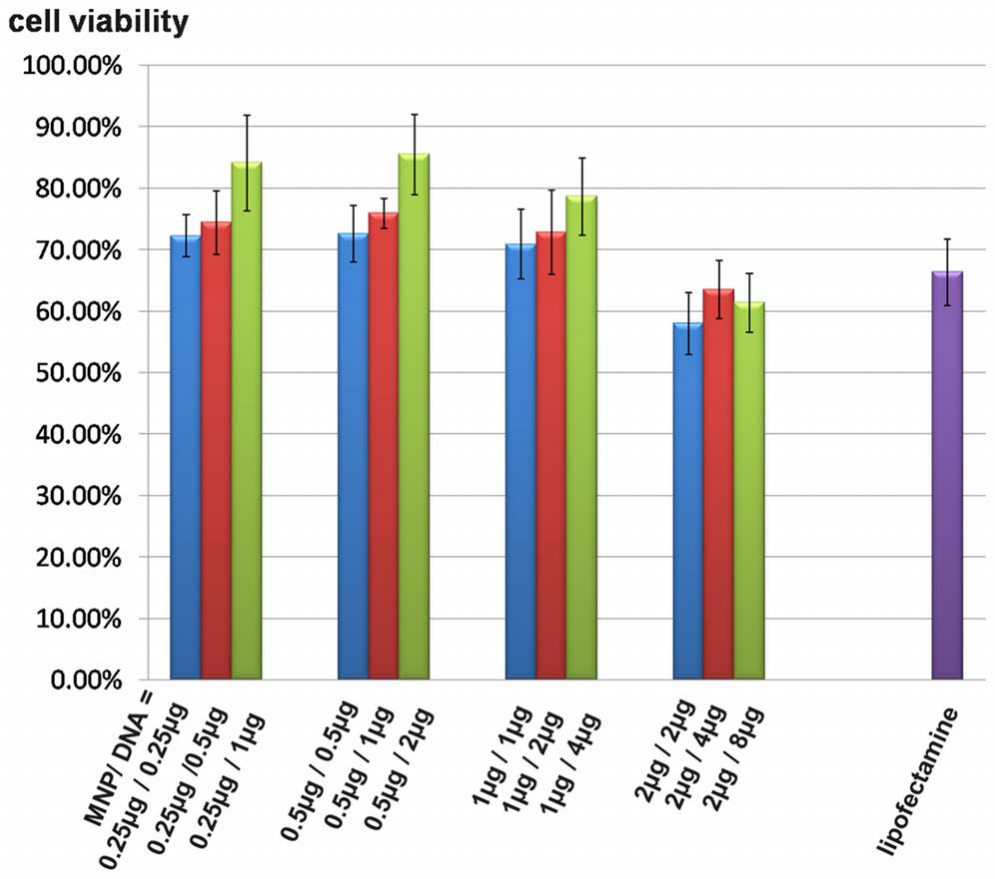
Cell viabilities of magnetofection and liposome transfection.

The cytotoxicity of MNPs was evaluated by MTT test. Cells were incubated with MNP/DNA complexes in a series of mass ratios. As shown in Fig. 9, cell viabilities over 70% with the concentrations of MNPs at 0.25 µg, 0.5 µg, 1 µg for 10^6^ cells, indicating the low cytotoxicity of MNPs at these concentrations. The survival rate of cells treated with lipofectamine was 66.3%, suggesting that MNPs had better biocompatibility than lipofectamine as gene vector. The survival rate of cells reduced with increased MNPs volume. In addition, MNP/DNA ratio also influenced cell viability and the increased DNA concentration is favorable to reducing the cytotoxicity.

### 3.8 Intracellular physical traces of magnetic nanoparticles

**Figure 10:**
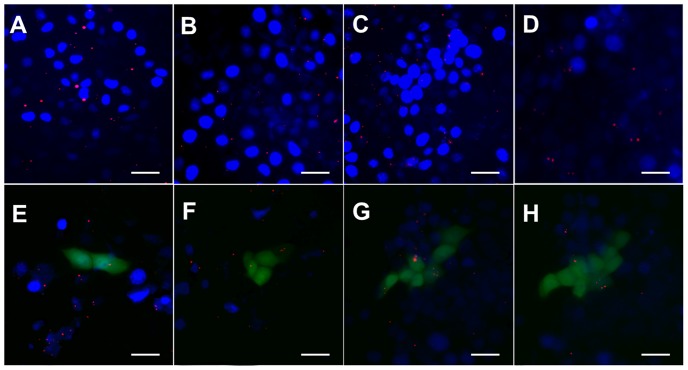
Fluorescence images of intracellular physical traces of red fluorescence MNPs and the expression of GFP in PK15 cells with time. A) 2 h, MNPs move though the cell membrane. B) 6 h, most MNPs are in the cytoplasm. C) 12 h, MNPs transfer from the cytoplasm to the nucleus. D) 18 h, most MNPs are inside the nucleus. E) 24 h, cells express GFP and MNPs shift back to the cytoplasm. F) and G) 36–48 h, MNPs gradually excrete out the cells and release in culture medium. H) 72 h, most MNPs escape from cells (scale bar  = 50 µm).

The intracellular physical traces of MNPs were tracked temporally and spatially via fluorescent imaging (Fig. 10). MNPs were taken into cells with time. Red fluorescence MNPs were detected on the surfaces of the cell membrane after 2 h (Fig. 10A). MNPs gradually moved to the cytoplasm after 6 h (Fig. 10B), and most MNPs were inside the nucleus after 12 h–18 h (Fig. 10C, D). The infected cells successfully expressed EGFP gene after 24 h, meanwhile, MNPs began to move from inside towards outside (Fig. 10E). MNPs gradually escaped from cells and only a few nanoparticles stayed inside cells after 72 h (Fig. 10H). Tracing results illustrated that MNPs directionally delivered and released exogenous DNA into the nucleus, resulting in the successful expression of exogenous gene.

## Conclusions

In summary, we characterized and evaluated the performances of MNP/DNA complexes as gene delivery system. MNP/DNA complexes were prepared by loading plasmid DNA using MNPs as gene carrier.

MNPs show superior properties in concentrating and protecting the exogenous nucleic acids. Morphology characterizations clearly demonstrate the assembling mechanism of MNP/DNA complexes that DNA molecules are adsorbed on the surface of MNPs. MNPs are effectively loaded with DNA via electrostatic attraction, electrostatically aggregation induces condensation of DNA, and the well-condensed structure in MNP/DNA complexes can protect DNA against enzymatic degradation.

In addition, MNPs are biocompatible with low cytotoxicity. Typical pathways of endocytosis and exocytosis are observed in the intracellular trail of fluorescent MNPs. MNPs showed internalize by cellular uptake firstly, and then transfer from the cytoplasm to the nucleus. Finally, MNPs were excreted out of cells after finishing the transfection process.

Importantly, MNPs can effectively transfer exogenous gene into mammalian somatic cells with highly stable gene expressions. Furthermore, various morphologies of MNP/DNA complexes can be formed with different MNP/DNA ratios, which influence their biological functions, and further result in different performances in cytotoxicity and dose–response relationships. Our study gives an insight on the morphologies and functionalization of MNPs gene delivery system, and provides an important experimental basis for the application of MNPs for effective magnetofection.
